# Simultaneous evaluation of EGFR, ALK, and PD-L1 in lung adenocarcinomas: the largest single-center experience from southern Brazil

**DOI:** 10.1590/1678-4685-GMB-2025-0150

**Published:** 2026-06-26

**Authors:** Daniel Cury Ogata, Matheus Mariotti Daniel, Bruno Vargas, Kauí Lebarbenchon, Guilherme Zappelini Zanette, Rafael Simas, Luciana Depiere Lanzarin, Fernanda Herbstrith de Sampaio

**Affiliations:** 1 Universidade do Vale do Itajaí (UNIVALI), Escola de Ciências da Saúde, Itajaí, SC, Brazil.; 2 Johns Hopkins University School of Medicine, The Sidney Kimmel Comprehensive Cancer Center, Baltimore, USA.

**Keywords:** Non-small cell lung cancer, lung adenocarcinoma, PD-L1 protein, EGFR genes, anaplastic lymphoma kinase

## Abstract

Lung cancer is a highly prevalent disease and the leading cause of cancer-related deaths worldwide. Among non-small cell lung carcinomas (NSCLC), adenocarcinoma is one of the most common subtypes. This retrospective study aimed to analyze the prevalence of epidermal growth factor receptor (EGFR) mutations and programmed death-ligand 1 (PD-L1) expression in patients with confirmed lung adenocarcinoma, based on pathology reports from 2019 to 2024. Patients were assessed by sex, age, PD-L1 expression, and presence of EGFR and ALK mutations. A total of 895 patients were included, mostly male, with an average age of 65.85 years. EGFR mutations were identified in 24.4% of the cases, predominantly exon 19 deletions (50.2%), with women accounting for 70.3% of those mutations. PD-L1 expression, determined by the tumor proportion score (TPS), was high (TPS ≥ 50%) in 28.9%, low (1 ≤ TPS ≤ 49%) in 26.3%, and absent (TPS < 1%) in 44.8% of patients. ALK mutations were found in 5.1% of cases, mostly among younger individuals. Findings on EGFR mutations were consistent with the national and international literature. However, PD-L1 expression rates were higher than those typically reported in Brazilian studies, highlighting regional variation in biomarker prevalence.

Lung cancer, the major cause of cancer-related deaths worldwide, is classified as small-cell lung cancer (SCLC) and non-small-cell lung cancer (NSCLC). NSCLC represents about 85% of the total number of pulmonary cancers, further subdivided into three groups: i) lung squamous cell carcinoma; ii) large cell carcinoma; and iii) lung adenocarcinoma, which is the most common histological subtype of primary lung cancer (Robles Gómez *et al*., 2024, Desharnais et al., 2025).

Although smoking is still the main risk factor for lung cancer development, infectious diseases, occupational hazards, radon exposure, and genetic susceptibility have also been identified as factors in never-smoker patients (Sharma et al., 2024). A study published in 2012 (Coté et al., 2012), based on the International Lung Cancer Consortium, described a 1.5-fold increase in the risk of lung cancer in first-degree relatives. These observations reinforce the necessity of a better understanding of the genetics behind the tumor.

Oncogenic drivers, such as mutation of the epidermal growth factor receptor (EGFR) and anaplastic lymphoma kinase (ALK) gene fusion, are associated with lung adenocarcinoma (Shukuya and Takahashi, 2019). Frequency of EGFR mutation is important to characterize advanced NSCLC in different classes (Zhang et al., 2024, Bearz et al., 2025). Another important feature is the expression of the programmed cell death ligand-1 (PD-L1) on the cell surface of the tumor. The expression of this biomarker inactivates T lymphocytes, resulting in tumor cell growth (Nawas et al., 2023). All these observations are important to determine optimal therapeutic strategy. In the present study, we describe the profile of patients with adenocarcinoma lung cancer diagnosis from a center in the south of Brazil, focusing on the presence of EGFR mutation and its association with ALK translocation.

The present study is a retrospective epidemiological study of patients from southern Brazil diagnosed with non-small cell lung cancer (NSCLC), whose pathology reports were issued in Itajaí, Santa Catarina, between 2019 and 2024. The research project was approved by the Research Ethics Committee of the University of Vale do Itajaí (UNIVALI), Santa Catarina, in accordance with the ethical standards for human research established by the National Committee of Research Ethics (CONEP).

The study population is comprised of patients diagnosed with lung adenocarcinoma who underwent testing for epidermal growth factor receptor (EGFR) mutation, anaplastic lymphoma kinase (ALK) rearrangement, and programmed death-ligand 1 (PD-L1) expression between 2019 and 2024. EGFR mutations were evaluated using real-time polymerase chain reaction (RT-PCR), while ALK-1 and PD-L1 expression were assessed via immunohistochemistry. All evaluations were performed in-house and independently verified by two pathologists. Patients who underwent only one or none of the proposed tests, those with other histological types and subtypes of cancer, and inconclusive reports were excluded from the study.

Patients were evaluated based on sex, age, level of PD-L1 expression, and the presence of mutations in EGFR and ALK genes. Disease stage and time since diagnosis were not considered.

Patient age was categorized as follows: under 40 years, between 40 and 60 years, and 60 years or older. The PD-L1 expression data were then subdivided into three groups: high expression (≥ 50% of cells), low expression (1 ≤ expression < 50%), or no evidence of expression (< 1%). The data concerning EGFR mutations were meticulously categorized according to the specific mutation type. ALK translocations were methodically classified as either positive or negative.

A comparison of the variables was conducted using Pearson’s chi-square test with Yates’ correction. The significance level of 0.05 was adopted for the statistical analysis. Due to the presence of multiple comparisons within each group, the p-value threshold was adjusted using the Šidák correction to reinforce statistical significance.

The study comprised 895 participants who met the inclusion criteria. Ten patients were excluded due to inconclusive results, and an additional seven were excluded for failure to complete all examinations specified in the study protocol. The demographic composition of the cohort was as follows: 50% of the participants were male, with an average age of 65.85 years, and 74.9% of the participants were over 60 years of age. These data are summarized in Table 1.

**Table 1 -:** PD-L1 expression correlated with age, sex, histological type, ALK translocation, and EGFR mutation.

Characteristics (%)	N	PD-L1 High Expression (≥50%)	PD-L1 Low Expression (1%-49%)	PD-L1 No Expression (<1%)	
Total Sample	895	259 (28.9)	235 (26.3)	401 (44.8)	
Age group					p = 0.2310
<40 years	27	9 (33.3)	10 (37.0)	8 (29.7)	
40 - 60 years	197	52 (26.4)	49 (24.9)	96 (48.7)	
>60 years	671	198 (29.5)	176 (26.2)	297 (44.3)	
Sex					p = 0.6507
Male	452	122 (27.0)	113 (25.0)	217 (48.0)	
Female	443	137(31.0)	122 (27.5)	184 (41.5)	
ALK Translocation					p = 0.3224
Positive	46	15 (32.6)	15 (32.6)	16 (34.8)	
Negative	632	194 (30.7)	154 (24.3)	284 (45.0)	
Mutation EGFR					p = 0.04461
Mutated	219	51 (23.3)	67 (30.6)	101 (46.1)	
Wild-type	678	209 (30.8)	169 (24.9)	300 (44.3)	

With regard to the entire sample, PD-L1 expression was detected in 55.2% of cases (n = 494). The stratification of PD-L1 expression was determined by the tumor proportion score (TPS), and the categorization is outlined below: The prevalence of the condition was found to be 1% in 401 patients (44.8%), 1-49% in 235 patients (26.3%), and ≥50% in 259 patients (28.9%). The results of the study demonstrated that PD-L1 expression was not associated with sex or age, as presented in Table 1.

In cases of lung adenocarcinoma, the presence of ALK-positive status did not demonstrate a correlation with PD-L1 expression. However, a negative association between mutated epidermal growth factor receptor (EGFR) and programmed death-ligand 1 (PD-L1) expression was observed (p < 0.05), though this was not confirmed after applying the Šidák correction.

A comprehensive analysis of 897 samples revealed the presence of mutations in 24.4% of cases. Two patients (0.9%) exhibited double mutations, thereby elucidating the discrepancy between the number of individuals (n = 895) and the number of EGFR analyses (n = 897). The cases under consideration both occurred in female patients over the age of 60 and included exon 20 (T790M) mutations. One of these cases was associated with an exon 19 deletion, while the other was associated with the L858R mutation.

The most prevalent mutation was exon 19 deletion (50.2%), followed by L858R (34.7%). As demonstrated in Table 2, less prevalent mutations encompassed G719A/C/S, exon 20 insertion, S768I, L861Q, and T790M.

A significant association was identified between EGFR mutations and female patients (p < 0.0001), accounting for approximately 70% of the mutated sample. Furthermore, a higher incidence of mutations was observed in younger patients (aged <60 years) (p = 0.0007), along with an age-related correlation with mutation type. Exon 19 deletions decreased in prevalence with age, while L858R mutations increased (p < 0.0001), as demonstrated in Table 2.

**Table 2 -:** EGFR mutation types by age and sex.

Characteristics	N	Exon 19 Deletion	Exon 20 Insertion	S768I	L858R	L861Q	G719A/C/S	T790M
EGFR mutated	219	110 (50.2)	11 (5.0)	5 (2.3)	76 (34.7)	3 (1.4)	12 (5.5)	2 (0.9)
Age Group - *p* < 0,0001								
<40 years	8	6 (75.0)	1 (12.5)	0	1 (12.5)	0	0	0 (0.0)
40 - 60 years	63	32 (50.8)	4 (6.3)	3 (4.8)	16 (25.4)	2 (3.2)	6 (9.5)	0 (0.0)
>60 years	148	72 (48.6)	6 (4.0)	2 (1.3)	59 (39.9)	1 (0.7)	6 (4.1)	2 (1.4)
Sex - p < 0,0001								
Male	65	37 (56.9)	5 (7.7)	0 (0.0)	20 (30.8)	0 (0.0)	3 (4.6)	0 (0.0)
Female	154	73 (47.4)	6 (3.9)	5 (3.2)	56 (36.3)	3 (1.9)	9 (5.8)	2 (1.3)

ALK translocation was positive in 46 of the 678 patients who were tested. It is estimated that patients with epidermal growth factor receptor (EGFR) mutations do not present an anaplastic lymphoma kinase (ALK) rearrangement. With high statistical significance (p < 0.0001), ALK rearrangement was found to be more prevalent in younger patients, with no correlation found with sex. The entirety of the aforementioned information is presented in Table 3.

**Table 3 -:** ALK translocation in patients by age and sex.

Characteristics	N	Positive	Negative	
Adenocarcinoma	895	46 (5.1)	849 (94.9)	
Age Group				p < 0.0001
<40 years	27	7 (25.9)	20 (74.1)	
40 - 60 years	197	14 (7.1)	183 (92.9)	
>60 years	671	25 (3.7)	646 (96.3)	
Sex				p = 0.2528
Male	452	22 (4.9)	430 (95.1)	
Female	443	24 (5.4)	419 (94.6)	

The present study identifies different distributions of PD-L1 protein expression compared to other established national studies. An epidemiological study conducted in the Northeast region of Brazil found that 18.2% of 137 patients with adenocarcinoma had high PD-L1 expression, 32.7% had low expression, and 49.5% of cases had no protein expression (Oliveira et al., 2019). When the results of that study are compared with those of the present sample, the finding that approximately one-third of the subjects in our sample had TPS ≥ 50% becomes more relevant. However, it should be noted that the low expression levels observed in the present study are lower than those found in Northeast Brazil.

The relationship between PD-L1 expression in adenocarcinoma and the presence of EGFR gene mutations is a contentious issue across various studies. The findings of Takada et al. (2018), which included 441 patients in Japan, suggest a significant relationship in which high PD-L1 expression is rarely associated with any type of EGFR mutation. From an alternative standpoint, the study by D’Incecco et al. (2015) - which comprised 125 patients from three Italian medical centers - demonstrated a close association with the presence of EGFR gene mutations. The findings of the present study indicate a negative correlation between mutated EGFR and PD-L1 expression, with a statistically significant p-value. This contradicts the conclusion reached by Takada *et al*. (2018). However, subsequent to the implementation of the Šidák correction, this value does not attain the level required to express strong evidence.

At the global level, the cohort by Pasello *et al*. (2022) - which analyzed 225 patients with lung adenocarcinoma at different stages of progression - focused on the group with the EGFR mutation (20%) and found a significant correlation with female sex and age below 70 years. A study conducted on a national scale by Mascarenhas et al. (2021) revealed that, among cases of EGFR mutation, 55% of the subjects were female, constituting a total sample of 513 patients. The findings of the current analysis are corroborated by the results of both studies.

In 2023, the most extensive Brazilian study on the subject of EGFR mutational status in NSCLC, conducted by Montella et al. (2023), evaluated the presence of EGFR mutations in a total of 7,413 patients over the period from 2013 to 2017, employing a range of methodologies. The study’s findings revealed that 24.2% of the sample population exhibited some form of EGFR gene mutation, either as a single mutation (22.7%) or as multiple mutations (1.5%). Exon 19 deletion was the most prevalent, found in 12.8% of participants, followed by the L858R mutation in exon 21 (6.9%) and exon 20 insertion (1.6%). A total of 4% of the sample was accounted for by less common mutations, including T790M, G719A/C/S, S768I, and L861Q. The results demonstrate notable parallels with the present study, given that the combined prevalence of exon 19 deletion and L858R mutation accounted for 77% of mutated cases in the national study and 84.3% in the southern Brazilian population represented in this study. The uncommon T790M mutation was not identified in isolation in the present study, but rather in association with other mutations, which differs from the aforementioned findings.

The most prevalent complex mutations identified by Montella et al. (2023) were exon 19 deletion + T790M (exon 20) in 2.3% of mutated cases, and L858R + T790M (exon 20) in 1.2% of cases. A lower identification rate of such mutations was observed in the present sample.

The primary conclusion of the northeastern Brazilian study by Oliveira et al. (2019) pertains to the high prevalence of ALK positivity (10.4%) observed in their sample, which significantly deviates from the prevalence documented in our sample (5.1%). The ALK rearrangement was present in only 3.6% of cases in the southern and southeastern Brazilian study by Gelatti *et al*. (2020). It is evident that the available national data are not consistent with each other, thereby corroborating the notion of population heterogeneity. A study by Dietel et al. (2018) found that, on a global scale, ALK translocation is present in 5% of lung adenocarcinoma cases. This result is consistent with the findings of the present study.

In light of the findings, the analysis of the prevalence of EGFR mutation and PD-L1 expression in patients diagnosed with lung adenocarcinoma in a population from southern Brazil suggests that the prevalence of the gene mutation and protein expression aligns with both global and national literature regarding EGFR mutations. However, the analysis indicates higher PD-L1 expression rates when compared to national data.

The present study has identified that exon 19 deletions are the most prevalent of the EGFR gene mutations, with strong statistical evidence supporting this finding. Furthermore, the analysis indicates that female patients and those under 60 years of age are the primary demographic affected by these mutations. Conversely, ALK translocation manifested with greater frequency in patients under 40 years of age, exhibiting statistical significance, though no substantial association was observed with regard to sex.

In terms of statistical disparities observed among studies from disparate Brazilian regions, it is imperative to undertake a comprehensive characterization of this demographic to facilitate a more precise epidemiological comprehension of Brazil. On a global scale, the results were similar, supporting the notion that the heterogeneous sample used may serve as a representative indicator of the global population.

## Data Availability

All data are stored and are available from the corresponding author upon reasonable request.
